# Supplementary figures and images for: The cuticle inward barrier in Drosophila melanogaster is shaped by mitochondrial and nuclear genotypes and a sex-specific effect of diet
Source: PeerJ. 2019 Oct 4;7:e7802. doi: 10.7717/peerj.7802 (PMC6779114; doi:10.7717/peerj.7802)

Individuals – PCA

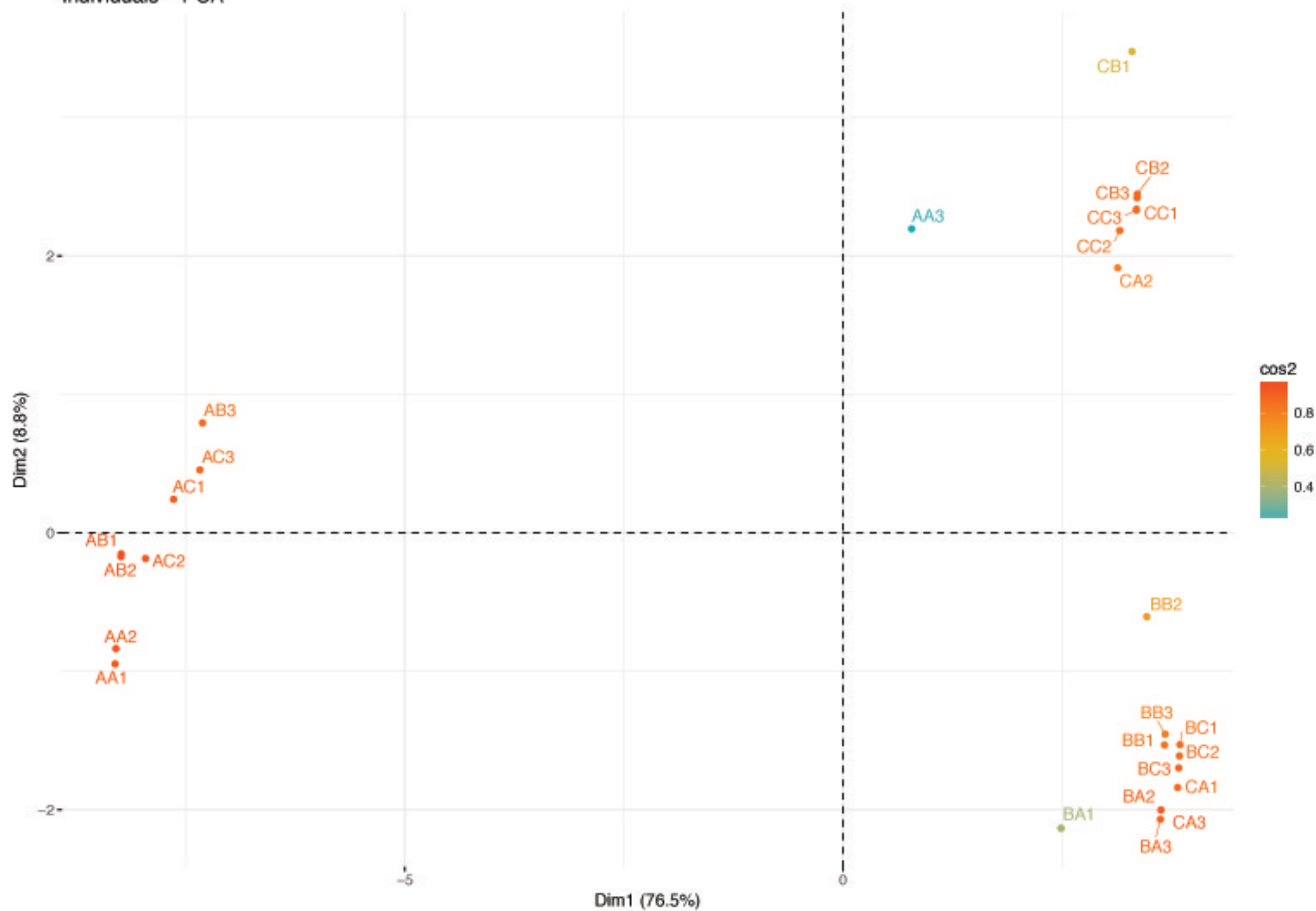

Supplement: Figure S1 — The mitochondrial genotypes were grouped based on the result by a PCA. We distinguish three groups. The mitochondrial genotypes AA1, AA2, AB1-3 and AC1-3 were grouped and named type ‘A’, the mitochondrial genotypes BA1-3, BB1-3, BC1-3, CA1 and CA3 were grouped and named type ‘B’ ,the mitochondrial genotypes AA3, CA2, CB1-3 and CC1-3 were grouped and named type ‘C’. [file peerj-07-7802-s001.pdf]

# Frequencies of wing patterns

no spot stained      rear spot stained      both spots stained

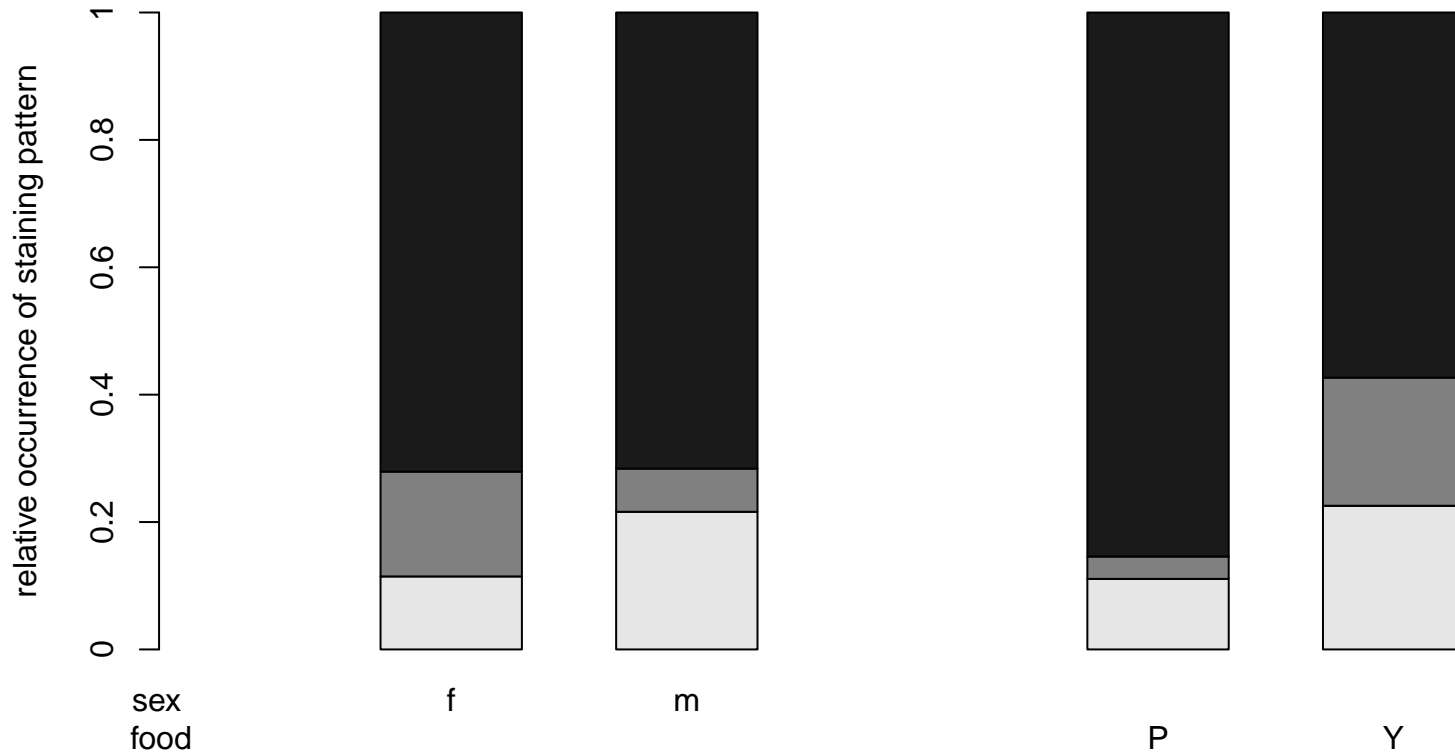

Supplement: Figure S2 — Relative frequencies by sex are: male no staining: 21.61%, rear area stained: 6.79%, both areas stained: 71.61%; female no staining: 11.46%, rear area stained: 16.45%, both stained: 72.09%. Staining frequencies by food: plant food no staining: 11.07%, rear area stained: 3.51%, both areas stained: 85.41%; yeast food no staining: 22.56%, rear area stained: 20.11%, both areas stained: 57.33%. [file peerj-07-7802-s002.pdf]
