# Supplementary material for: The cuticle inward barrier in Drosophila melanogaster is shaped by mitochondrial and nuclear genotypes and a sex-specific effect of diet
Source: PeerJ. 2019 Oct 4;7:e7802. doi: 10.7717/peerj.7802 (PMC6779114; doi:10.7717/peerj.7802)
Supplement: Table S1 — ∘ ∘ indicates that none of the two areas was stained, ∘ • indicates that the rear wing area was stained and • • indicates that both areas on the wing were stained. Empty cells indicate that the specific combination of all four factors did not occur in our experiment after recording of the mitochondrial genotypes to five levels based on the sequence data. [file peerj-07-7802-s003.docx]

|  |  | male | | | | | | | | | female | | | | | | | | |
| --- | --- | --- | --- | --- | --- | --- | --- | --- | --- | --- | --- | --- | --- | --- | --- | --- | --- | --- | --- |
| nuclear |  | A | | | B | | | C | | | A | | | B | | | C | | |
|  | mitochondrial | ∘∘ | ∘• | •• | ∘∘ | ∘• | •• | ∘∘ | ∘• | •• | ∘∘ | ∘• | •• | ∘∘ | ∘• | •• | ∘∘ | ∘• | •• |
|  | A | 10 | 2 | 8 | 0 | 0 | 34 | 2 | 1 | 16 | 10 | 2 | 8 | 2 | 0 | 32 | 1 | 1 | 0 |
| plant | B | 13 | 2 | 38 | 0 | 0 | 35 | 4 | 0 | 28 | 9 | 8 | 29 | 1 | 1 | 31 | 0 | 0 | 31 |
|  | C | 5 | 0 | 18 | 1 | 0 | 28 | 0 | 1 | 32 | 5 | 0 | 15 | 1 | 1 | 26 | 0 | 1 | 33 |
|  | A | 19 | 1 | 0 | 1 | 0 | 18 | 9 | 8 | 13 | 7 | 5 | 4 | 1 | 1 | 18 | 1 | 14 | 15 |
| yeast | B | 31 | 4 | 15 | 0 | 0 | 30 | 4 | 13 | 13 | 17 | 24 | 9 | 0 | 1 | 28 | 2 | 14 | 6 |
|  | C | 12 | 2 | 6 | 4 | 0 | 26 | 7 | 4 | 18 | 5 | 1 | 5 | 0 | 0 | 30 | 0 | 6 | 24 |
